# Supplementary material for: EnzML: multi-label prediction of enzyme classes using InterPro signatures
Source: BMC Bioinformatics. 2012 Apr 25;13:61. doi: 10.1186/1471-2105-13-61 (PMC3483700; doi:10.1186/1471-2105-13-61)
Supplement: Addtional file 5 — The Java code to format the data files, evaluate and predict. The file enzml_java_code.tar.gz contains the Java code used to format database data to ARFF and XML formats, to execute cross and train-test (jackknife) evaluations and to record evaluation results to database. More information is included in the readme.txt file and the Javadoc files. The code can be used with a MySQL database. To use a different database software, other JDBC drivers might be required. [file 1471-2105-13-61-S5.gz › java_code/enzml2011/doc/uk/ac/ed/inf/taweka/DataSetGenerator.html]

DataSetGenerator


---


|  |  |  |
| --- | --- | --- |
| **Overview** \| **Package** \| **Class** \| **Use** \| **Tree** \| **Deprecated** \| **Index** \| **Help** | |  |
| **PREV CLASS**   **NEXT CLASS** | **FRAMES**    **NO FRAMES**     **All Classes** |  |
| SUMMARY: NESTED \| FIELD \| CONSTR \| METHOD | DETAIL: FIELD \| CONSTR \| METHOD |  |


---


## uk.ac.ed.inf.taweka Class DataSetGenerator

```
java.lang.Object
  uk.ac.ed.inf.taweka.DataSetGenerator
```

**Direct Known Subclasses:**: MulanDataSetGenerator

---

``` public class DataSetGenerator extends java.lang.Object ```

Generates and fills the Weka instances

**Version:**
:   3 Sep 2010

**Author:**
:   Luna De Ferrari luna.deferrari-at-ed.ac.uk

---

**Constructor Summary**

`DataSetGenerator(DataSetManager manager,
uk.ac.ed.inf.utils.OneToManyMap instanceAttributeMap,
uk.ac.ed.inf.utils.OneToManyMap instanceClassMap)`


| **Method Summary** | |
| --- | --- |
| `void` | `fillAndWriteInstances()` |
| `java.util.ArrayList<weka.core.Attribute>` | `getAttributes()` |
| `AttributeFactory` | `getAttributesFactory()` |
| `java.lang.String` | `getClassName()` |
| `uk.ac.ed.inf.utils.OneToManyMap` | `getInstanceAttributeMap()` |
| `uk.ac.ed.inf.utils.OneToManyMap` | `getInstanceClassMap()` |
| `weka.core.Instances` | `getInstances()` |
| `InstancesFiller` | `getInstancesFactory()` |
| `java.util.Vector<java.lang.String>` | `getInstancesNames()` |
| `DataSetManager` | `getManager()` |
| `int` | `getNumberOfAttributes()` |
| `int` | `getNumberOfClassValues()` |
| `int` | `getNumberOfInstances()` |
| `void` | `setInstancesNames()` |

**Methods inherited from class java.lang.Object**

`equals, getClass, hashCode, notify, notifyAll, toString, wait, wait, wait`

**Constructor Detail**

### DataSetGenerator

```
public DataSetGenerator(DataSetManager manager,
                        uk.ac.ed.inf.utils.OneToManyMap instanceAttributeMap,
                        uk.ac.ed.inf.utils.OneToManyMap instanceClassMap)
```


**Method Detail**

### fillAndWriteInstances

```
public void fillAndWriteInstances()
```

---


### getAttributes

```
public java.util.ArrayList<weka.core.Attribute> getAttributes()
```

---


### getAttributesFactory

```
public AttributeFactory getAttributesFactory()
```

---


### getClassName

```
public java.lang.String getClassName()
```

---


### getInstanceAttributeMap

```
public uk.ac.ed.inf.utils.OneToManyMap getInstanceAttributeMap()
```

---


### getInstanceClassMap

```
public uk.ac.ed.inf.utils.OneToManyMap getInstanceClassMap()
```

---


### getInstances

```
public weka.core.Instances getInstances()
```

---


### getInstancesFactory

```
public InstancesFiller getInstancesFactory()
```

---


### getInstancesNames

```
public java.util.Vector<java.lang.String> getInstancesNames()
```

---


### getManager

```
public DataSetManager getManager()
```

---


### getNumberOfAttributes

```
public int getNumberOfAttributes()
```

---


### getNumberOfClassValues

```
public int getNumberOfClassValues()
```

---


### getNumberOfInstances

```
public int getNumberOfInstances()
```

---


### setInstancesNames

```
public void setInstancesNames()
```


---


|  |  |  |
| --- | --- | --- |
| **Overview** \| **Package** \| **Class** \| **Use** \| **Tree** \| **Deprecated** \| **Index** \| **Help** | |  |
| **PREV CLASS**   **NEXT CLASS** | **FRAMES**    **NO FRAMES**     **All Classes** |  |
| SUMMARY: NESTED \| FIELD \| CONSTR \| METHOD | DETAIL: FIELD \| CONSTR \| METHOD |  |


---
